# The bacterial interactions in the nasopharynx of children receiving adenoidectomy

**DOI:** 10.7603/s40681-015-0006-9

**Published:** 2015-02-02

**Authors:** Hao-Xiang Chen, Chih-Ho Lai, Hui-Ying Hsu, Ju-Chun Huang, Hua-Shan Wu, Mao-Wang Ho, Ming-Hsui Tsai, Chia-Der Lin

**Affiliations:** 1 School of Medicine and Graduate Institute of Basic Medical Science, China Medical University, 404 Taichung, Taiwan; 2 Department of Nursing, Asia University, 413 Taichung, Taiwan; 3 Department of Laboratory Medicine, China Medical University Hospital, 404 Taichung, Taiwan; 4 Department of Internal Medicine, China Medical University Hospital, 404 Taichung, Taiwan; 5 Graduate Institute of Clinical Medical Science, China Medical University, 404 Taichung, Taiwan; 6 Department of Otolaryngology-Head and Neck Surgery, China Medical University Hospital, No. 2, Yuh-Der Road, 404 Taichung, Taiwan

**Keywords:** Adenoid;, Bacterial interactions;, *Haemophilus influenzae;*, *Staphylococcus aureus;*, *Streptococcus*, *pneumoniae*

## Abstract

*Staphylococcus aureus, Streptococcus pneumoniae*, and *Haemophilus influenzae* are the common pathogens that colonize in the nasopharynx of children. Polymicrobial interactions are thought to play an important role in different sites throughout the human body. However, there are currently very few studies that investigate the interactions between *S. aureus, S. pneumoniae*, and *H. influenzae* in the nasopharynx. We retrospectively analyzed the adenoid tissue culture from 269 children who received adenoidectomy. *S. aureus, S. pneumoniae*, and *H. influenzae* constituted the major microorganisms which were cultured from these adenoidectomies, at 23.4%, 21.6%, and 18.2%, respectively. *S. pneumoniae and H. influenzae* were the most prevalent in the preschool-aged children (3 < age ≤ 6), whereas *S. aureus* was more prevalent in infants and toddlers (age ≤ 3) and school-aged children (age > 6). Bacterial interference was found between *S. aureus* and *S. pneumoniae* and between *S. aureus* and *H. influenzae*, whereas there was an association found between *S. pneumoniae* and *H. influenzae*. The synergism and antagonism among these three species are investigated in the following paper, with the possible mechanisms involved in these interactions also discussed.

## 1. Introduction

As the adenoid is located at the crossroads of the upper respiratory tract, adjacent to the middle ear, paranasal sinuses and oropharynx, chronic adenoiditis has been associated with the pathologies of the neighboring structures, such as otitis media and sinusitis [1]. The adenoid can serve as a bacterial reservoir that contributes to chronic otolaryngologic infections in children, infections such as otitis media and paranasal sinusitis [2]. The most common nasopharyngeal microbes that are found in children include *S. aureus, S. pneumoniae*, and *H. influenzae* [3]. *S. pneumoniae* is frequently concomitant with nasopharyngeal illnesses [4], while *H. influenzae* is a common pathogen of acute otitis media [5]. *S. aureus* is associated with skin or respiratory tract diseases such as chronic adenoiditis and rhinosinusitis [6, 7]. The emergence of methicillinresistant *S. aureus* (MRSA) has become an important public health problem, both as a rising community pathogen and with respect to its potential impact on strategies for antibiotic therapy [7].

More than one microorganism is frequently found in the nasopharynx and polymicrobial interactions definitely exist in the nasopharynx [3, 8-11]. Some bacterial species may co-exist more often with other species (synergistic interactions), while other species may compete with one another (antagonistic interactions). For an example of the latter: competitive interaction has been reported between *S. pneumoniae* and *S. aureus* [9, 11]. However, a detailed description regarding the interactions between *S. aureus, S. pneumoniae*, and *H. influenzae* in the nasopharynx of children is still limited.

The purpose of this study was to analyze the nasopharyngeal colonizations by the bacterial species *S. aureus, S. pneumoniae*, and *H. influenzae* in children receiving adenoidectomy. The interactions among the bacterial species were evaluated to see whether the colonization status of one species influences the colonization of the other two species.

**Table 1:** The demography of the enrolled patients.

Characteristic	No. (%)
Age (years)^†^	
age ≤ 3	15 (5.6)
3 < age ≤ 6	138 (51.3)
6 < age ≤ 12	106 (39.4)
12 < age	10 (3.7)
Gender^¶^	
F	102 (37.9)
M	167 (62.1)
Bacteria present	
0	37 (13.8)
1	160 (59.5)
2	56 (20.8)
≥ 3	16 (6.0)

^†^Age (years) of children at the time of adenoidectomy.

^¶^F, female; M, male.

## 2. Patients and methods

### 2.1. Patient selection

This study was carried out between January 2002 and December 2012 and comprised patients who were examined for otorhinolaryngologic infections, including chronic otitis media, otitis media with effusion, chronic rhinosinusitis, chronic adenoiditis, and chronic tonsillitis as well as those who were clinically diagnosed with upper respiratory problems. During this period, 276 participants were enrolled in this study and underwent routine adenoidectomy surgery and had a bacterial culture of their nasopharynx taken. A total of 269 patients whose ages ranged from 1 to 18 years old were analyzed. There were 102 girls (37.9%) and 167 boys (62.1%). The patients enrolled in this study had completed a self-administered questionnaire by their parents prior to being enrolled.

### 2.2. Ethics statement

This study was specifically approved by the Institutional Review Board of the China Medical University Hospital (approval number: DMR98-IRB-123, Taichung, Taiwan).

### 2.3. Laboratory procedure and bacterial culture

Core tissues from adenoid specimens and pus swabs from patients’ noses were streaked across Tryptic soy agar (Becton- Dickinson, Franklin Lakes, NJ, USA) containing 5% sheep blood and incubated at 37°C for 18-24 h. Bacterial isolates were identified by a standard protocol using the BD PhoenixTM Automated Microbiology System (Becton-Dickinson) as described in our previous study [7].

### 2.4. Statistical analysis

The relationship of between-group comparisons was performed using a *Chi*-square test with Fisher’s exact test. The correlation of bacterial infections in two species was assessed by odd ratio (OR) analysis. Descriptive statistics were determined as the proportion for categorical variables with 95% confidence intervals (CI). Statistical analyses were carried out using the SPSS program (version 12.0; SPSS Inc., Chicago, IL, USA). A *P* value less than 0.01 was considered statistically significant.

**Fig. 1:**
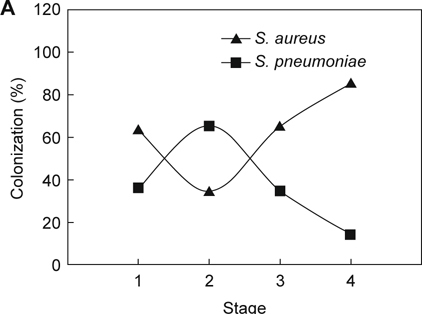

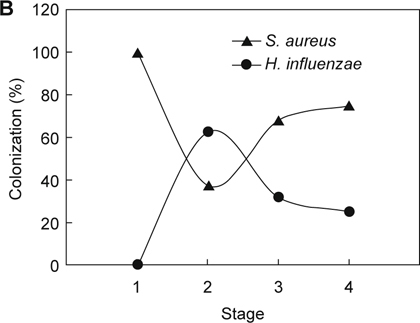

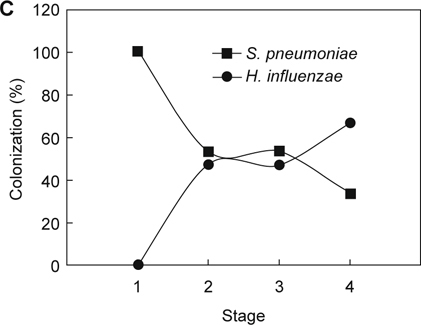
The age-related bacterial interactions in the nasopharynx of children receiving adenoidectomy. Patients who enrolled in this study were stratified into four age stages: Stage 1: age ≤ 3; Stage 2: 3 < age ≤ 6; Stage 3: 6 < age ≤ 12; Stage 4: age > 12. The colonization rates of each comparison between two bacterial species were determined and analyzed: (A) *S. aureus vs. S. pneumoniae*; (B) *S. aureus vs. H. influenzae*; (C) *S. pneumoniae vs. H. influenzae*.

**Table 2:** Inverse association of bacterial colonization in the nasopharynx of children.

				*S. aureus*			*S. pneumoniae*			*H. influenzae*	
		No. of isolates	No. (%)	OR^†^ (95% CI)	*P* value^¶^	No. (%)	OR (95% CI)	*P* value	No. (%)	OR (95% CI)	*P* value
*S. aureus*	Positive Negative	63 206									
*S. pneumoniae*	Positive Negative	58 211	6 (10.3) 57 (27.0)	0.31 (0.13-0.77 )	**0.008**		-				
*H. influenzae*	Positive Negative	49 220	3 (4.8) 46 (22.3)	0.17 (0.05-0.58 )	**0.002**	8 (13.8) 41 (19.4)	0.66 (0.29-1.51)	0.325		-	

^†^OR, odd ratio.

^¶^P value was determined from logistic regression model. A significant difference is indicated by a number in bold.

## 3. Results

### 3.1. Demography of the enrolled patients

To analyze the association between the carriages of three bacterial species, the young children with otorhinolaryngologic infections who visited China Medical University Hospital were enrolled in this study. The bacterial colonizations of the nasopharynx from children receiving adenoidectomies were then identified by a traditional culture method. Of all 276 participants, 269 patients < 18 years old were enrolled in this analysis. We then stratified the patients into four age stages: stage 1: age ≤ 3; Stage 2: 3 < age ≤ 6; Stage 3: 6 < age ≤ 12; Stage 4: age > 12. As shown in Table 1, there were 15, 138, 106, and 10 children in stages 1, 2, 3, and 4, respectively. Within this analysis, no bacterial species was isolated in 37 patients. However, at least one bacterial species of microbial colonization was cultured in 232 patients.

### 3.2. The associations of bacterial colonizations in children receiving adenoidectomy

To further analyze the associations between the pathogens which colonized in children who were receiving adenoidectomies, three bacterial species’ (*S. pneumoniae, S. aureus*, and *H. influenzae*) colonization in the aforementioned stages were analyzed. As shown in Figure 1A, higher rates of *S. aureus* colonized in patients belonging to stages 1, 3, and 4, *S. pneumoniae* colonization was lower at the same stages. Consistently, higher *S. aureus* colonization in stages 1, 3, and 4 were inversely related to *H. influenzae* infection in patients in these stages (Figure 1B). The bacterial carriage of *S. pneumoniae* was negatively associated with *H. influenzae* in stages 1 and 4. However, higher rates of *S. pneumoniae* colonized in stages 2 and 3, with higher rates of *H. influenzae* infection in the same stages (Figure 1C).

We then analyzed the correlation of bacterial infections in two species using logistic regression analysis. As shown in Table 2, *S. aureus, S. pneumoniae*, and *H. influenzae* constitute major microorganisms cultured from these adenoidectomies, at 23.4%, 21.6% to 18.2%, respectively. *S. aureus* colonization was significantly inversely associated with *S. pneumoniae* colonization and vice versa (OR = 0.31; 95% CI = 0.13-0.77, *P* = 0.008). Additionally, a negatively associated relationship was observed between *S. aureus and H. influenzae* (OR = 0.17; 95% CI = 0.05-0.58, P = 0.002). Although the bacterial carriages of *S. pneumoniae* was inversely associated with *H. influenzae* in stages 1 and 4 (Figure 1C), there was no significance (*P* = 0.325).

## 4. Discussion

In this study, we investigated the colonization of the nasopharynx in children receiving adenoidectomies using a traditional culture method. Our data showed that *S. aureus, S. pneumoniae*, and *H. influenzae* constitute major microorganisms cultured from the adenoidectomies at 23.4%, 21.6% to 18.2%, respectively (Table 2). These findings are similar to the previous study that *S. pneumoniae, H. influenzae, Moraxella catarrhalis*, and *S. aureus* are common nasopharyngeal colonizations found in children [3], though *M. catarrhalis* was not frequently isolated in our study. More than one microorganism was found in 26.8% of children receiving adenoidectomies, whereas no bacterium was cultured in 13.8% of the adenoid specimens (Table 1). The identification rate of microorganisms in this study may be under estimated. With the advances in microbial techniques such as real-time quantitative polymerase chain reaction (qPCR) technique, in future studies there may be more diverse microorganisms identified [12].

Our study showed that *S. pneumoniae* and *H. influenzae* were most prevalent in stage 2 (preschool period) (3 < age ≤ 6), whereas *S. aureus* was more prevalent in stage 1 (infant and toddler stage) (age ≤ 3) and stage 3&4 (age > 6). This result demonstrated that the prevalence of bacterial species may be varied in different age groups. Host factors including age may be important in the nasopharyngeal reservoir. Dynamic changes in nasopharyngeal microflora have been described [13]. Healthy children were generally colonized with relatively non-pathogenic microbes in their nasopharynx. *S. aureus* was frequently found in the infant period, with its carriage decreasing with a person’s age [14]. Conversely, *S. pneumoniae* and *H. influenzae* were not frequent isolates from the infant period [14] until the pre-school period [13]. However, carriage of potential respiratory pathogens such as *S. pneumoniae* and *H. influenzae* increased when purulent nasopharyngitis occurred [13]. In addition to age, other factors may influence the dynamic alterations of microbes in the nasopharynx. These factors include immunity, sibling number, crowding, season, use of antibiotics, acute respiratory tract infection, vaccine application, and passive smoking exposure [5, 15, 16].

Our study showed that *S. aureus* was inversely associated with *S. pneumoniae and H. influenzae*. This finding is consistent with several previous studies about the negative association of *S. aureus* with *S. pneumoniae* in the nasopharynx [10, 17-19]. Adaptive immunity has been proposed because such interference between *S. aureus* and *S. pneumoniae* was not shown in HIV-infected children [10, 17]. Free radicals may be another possible mechanism to explain this bacterial interference as the hydrogen peroxide produced by *S. pneumoniae* could elicit bactericidal activity toward *S. aureus* and prevent its colonization [19]. The interference between *S. aureus* and *H. influenzae* has also been shown [20]. Additionally, the different susceptibility in biofilm formation to environment such as hyaluronic acid has been proposed [21].

Contrary to the interference phenomenon between *S. aureus* and the other two species, an association was found between *S. pneumoniae* and *H. influenzae*, although the interaction was not significant. This result was similar to the previous epidemiologic observations [9, 10, 22, 23]. *H. influenzae* has been shown to promote the biofilm formation in *S. pneumoniae* [24]. However, similar free radical formation was also shown in vitro that the formation of hydrogen peroxide from the *S. pneumoniae* could inhibit the growth of *H. influenza* [25]. Another epidemiological observation showed an interference phenomenon between *S. pneumoniae* and *H. influenzae*, but the association could shift from negative to positive when *M. catarrhalis* appeared in the interaction [3]. These studies showed the complicated phenomenon in the microenvironment between bacterial synergism and antagonism.

This study presents the microbiological dynamics and the microbial interactions in the nasopharynx of children receiving adenoidectomies. A more complete understanding of how bacteria interact with each other may be important in future designs of preventive or therapeutic strategies. This may be important in the era of new vaccine or antimicrobial development, in which the influence of one specific bacterium may have a positive or negative impact on other species. Our study confirmed the interference between *S. aureus* and both *S. pneumoniae* and *H. influenzae*, and a possible association between *S. pneumoniae* and *H. influenzae*. The potential implications of targeting these interactions may serve as a route towards control of bacterial infections.

## 5. Conclusions

In this study, polymicrobial interactions were studied in the nasopharynxes of children who received adenoidectomies. Bacterial interference was found between *S. aureus* and *S. pneumoniae* and between *S. aureus* and *H. influenzae*, whereas, an association was found between *S. pneumoniae* and *H. influenzae*. These findings lead to the appreciation that many infections are polybacteria in nature, and that interactions between different microorganisms may contribute to disease progression and clinical outcomes.

## Acknowledgements

The authors thank Dr. Ming-Chei Maa for her valuable suggestions and editorial assistance. We also thank Biostatistics Center at China Medical University for the data analysis. This work was funded by the Ministry of Science and Technology (102-2314- B-039-023-MY2, 103-2633-B-039-001, and 103-2815-C-039- 062-B), Ministry of Health and Welfare Clinical Trial and Research Center of Excellence (MOHW104-TDU-B-212-113002), China Medical University (CMU102-ASIA-21 and DMR103- 024), and the Tomorrow Medicine Foundation.

### Declaration of interest

The authors declare no conflicts of interest for this work.
